# Eficacia del índice mandibular canino en la determinación del sexo de una población peruana: un estudio transversal

**DOI:** 10.15446/rsap.V25n3.105126

**Published:** 2023-05-01

**Authors:** Miguel Alvarado-Vicuña, Jaime Plasencia-Castillo, Paul M. Herrera-Plasencia, Gustavo Jiménez-Carreño

**Affiliations:** 1 MA: OD. Universidad César Vallejo. Piura, Perú. maralvarado@ucvvirtual.edu.pe Universidad César Vallejo Universidad César Vallejo Piura Peru maralvarado@ucvvirtual.edu.pe; 2 JP: OD. Ph. D. Criminalística. Universidad César Vallejo. Piura, Perú. juplasenciac@ucvvirtual.edu.pe Universidad César Vallejo Criminalística Universidad César Vallejo Piura Peru juplasenciac@ucvvirtual.edu.pe; 3 PH: OD. M. Sc. Estomatología. Ph. D. Criminalística. Universidad César Vallejo. Piura, Perú. pherrera@ucv.edu.pe Universidad César Vallejo Criminalística Universidad César Vallejo Piura Peru pherrera@ucv.edu.pe; 4 GJ: OD. Ph. D. Educación. Universidad César Vallejo. Piura, Perú. gajimenezc@ucvvirtual.edu.pe Universidad César Vallejo Universidad César Vallejo Piura Peru gajimenezc@ucvvirtual.edu.pe

**Keywords:** Odontología forense, odontometría, sexo *(fuente: DeCS, BIREME)*, Forensic dentistry, odontometry, sex *(source: MeSH, NLM)*

## Abstract

**Objetivos:**

Determinar la eficacia del índice mandibular del canino (IMC) en una población universitaria del norte del Perú.

**Material y Métodos:**

El estudio es descriptivo, observacional, transversal, prospectivo. La muestra estuvo constituida por 168 personas: 84 hombres y 84 mujeres, estudiantes de ciencias de la salud de una universidad privada de Piura (Perú), cuyas edades estuvieron comprendidas entre los 18 y los 35 años. Se elaboraron modelos de yeso y mediante el uso de un vernier digital calibrado se realizó la medición del ancho mesiodistal máximo del canino mandibular y la distancia intercanina. De la fracción de ambas magnitudes se obtuvo el IMC, el cual fue ajustado para la población estudiada. La eficacia para la estimación de sexo de este nuevo punto de corte fue estimada sobre la base del área bajo la curva ROC y el estadístico Hanley y McNei.

**Resultados:**

El nuevo IMC encontrado en nuestra población fue de 0,258. Tanto el ancho mesiodistal de canino mandibular como la distancia intercanina demostraron ser discriminantes de sexo (p < 0,05); no hubo diferencias significativas (p > 0,05) en el diámetro mesiodistal de los caninos derecho e izquierdo; es posible utilizar cualquiera de los dos.

**Conclusiones:**

La efectividad del IMC para la estimación del sexo con el punto de corte estimado para la población del norte del Perú es del 71,7 %, porcentaje que sirve para la toma de decisiones. Sin embargo, se recomienda complementar con otros medios odontológicos basados en odontometría y osteometría, puesto que la certeza no es absoluta.

## Abstract (en)

**Objectives:**

To determine the effectiveness of the Canine Mandibular Index (CMI) in a university population in northern Peru.

**Material and Methods:**

Descriptive, observational, cross-sectional and prospective studies. The sample consisted of 168 persons: 84 males and 84 females, students of health sciences from a private university in Piura (Peru), whose ages ranged from 18 to 35 years. Plaster models were made and, by using a calibrated digital vernier, the maximum mesiodistal width of the mandibular canine and the intercanine distance were measured. From the fraction of both magnitudes, the BMI was obtained, which was adjusted for the population studied. The efficacy for the sex estimation of this new cut point was estimated based on the AUC and Hanley and McNeil test.

**Results:**

The new CMI found in our population was 0.258. Both the mesiodistal width of mandibular canine and the intercanine distance were shown to be sex-discriminatory (p < 0.05); there were no significant differences (p > 0.05) in the mesiodistal diameter of the right and left canine, being possible to use either of the two.

**Conclusions:**

The effectiveness of the CMI for sex estimation, with the estimated cut-off point for the population of northern Peru is 71.7 °%, this percentage is used for decision making, however it is recommended to complement other dental means based on odontometry and osteometry, since the certainty is not absolute.

La identificación de cadáveres utilizando restos óseos y la dentadura es una práctica común en la antropología física, la odontología forense y la arqueología. La evaluación del sexo de tales restos es un paso importante en la construcción del perfil biológico de esqueletos no identificados, puesto que a partir de este se desarrollan los algoritmos para la estimación de los otros componentes, tales como: edad, raza o talla 1.

Son múltiples los estudios y los huesos utilizados para la estimación del sexo en cadáveres (fémur, húmero, escápula, clavícula, rótula, esternón, hioides, mandíbula); sin embargo, existe un amplio consenso de que el cráneo y la pelvis son las regiones más útiles para este fin 2,3. Mestekova *et al.*^4^ encontraron en una población francesa una precisión del 92,3 % en hombres y del 97,2 % en mujeres utilizando tomografías computarizadas y el método DSP (probabilidad para el diagnóstico de sexo) en pelvis. Asimismo, Small *et al.*^5^, mediante funciones discriminantes de 990 distancias intercraneales, obtuvieron una precisión de estimación global del sexo del 88,2 %.

Nuestra sociedad actual enfrenta muertes repentinas e inesperadas, producto de desastres naturales (terremotos, tsunamis, huracanes, entre otros) y desastres antrópicos (incendios, accidentes aéreos y ferroviarios, terrorismo y delincuencia común), donde los huesos craneales se fragmentan y las partes del cuerpo se descomponen o mutilan, lo cual hace difícil la identificación 6. En estas condiciones, la dentición actúa como un complemento útil en la identificación, porque es el tejido más estable y más duro del cuerpo, además de soportar la sumersión y las temperaturas reportadas experimentalmente de hasta 600° C y en siniestros de 1000° C 7.

El dimorfismo sexual se refiere a las diferencias de tamaño, estatura y apariencia entre hombres y mujeres que se pueden aplicar a la identificación dental, porque no hay dos bocas iguales 8. Garn *et al.*^9^ estudiaron la magnitud de dicho dimorfismo en la población caucásica y concluyeron que tal magnitud en los dientes caninos variaba entre los diferentes grupos étnicos. Rao *et al.*^10^ estudiaron una población del sur de India y concluyeron que el 84,3 % de los hombres y el 87,5 % de las mujeres podrían ser discriminados correctamente con respecto al sexo. El punto de corte discriminante se obtiene de la razón entre el diámetro mesiodistal de la corona del canino inferior y la distancia intercanina mandibular, establecido por los autores en 0,274; valores por encima de este pertenecen al sexo masculino, mientras que valores inferiores corresponden al sexo femenino (Figura 1) 6.

**Figura 1:**
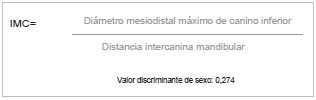
Algoritmo del índice mandibular del canino

Fuente: Rao et al. 10^).^

Los caninos mandibulares no solo están menos expuestos a placa, cálculo, abrasión por cepillado o carga oclusal abundante que otros dientes, sino que también se ven menos afectados por la enfermedad periodontal y, por lo tanto, se prolonga su permanencia en la boca, por lo cual son considerados dientes clave para la identificación del sexo.

Diferentes investigadores 2,3,6,11-14 han mostrado aplicaciones prometedoras para la identificación del sexo en situaciones arqueológicas y forenses, por medio del índice mandibular del canino (IMC), con tasas de éxito que van del 63 % al 85,5 %, y han resaltado que es simple, confiable, económico y fácil de realizar. Por otro lado, existen investigadores que rechazan su eficacia y aplicación 15,16.

En la actualidad se carece de estudios de referencia para la predicción del sexo utilizando datos odontométricos en el norte del Perú. De acuerdo con Garn *et al.*^9^, los grupos étnicos presentan diferente dimorfismo sexual, por ende, este estudio tiene como objetivo determinar la eficacia del IMC de Rao *et al.*^10^ en una población universitaria del norte del Perú, además de establecer la importancia de los discriminantes del IMC (diámetro mesiodistal y distancia intercanina) en el dimorfismo sexual, y, por último, determinar el IMC para la población estudiada y la eficacia de dicho índice.

## MATERIAL Y MÉTODOS

El estudio es descriptivo, observacional, transversal, prospectivo. La muestra fue escogida por conveniencia y consistió en 168 personas: 84 hombres y 84 mujeres, estudiantes de ciencias de la salud de una universidad privada de Piura (Perú), cuyas edades estuvieron comprendidas entre 18 y 35 años. Fueron excluidos los estudiantes con historia de tratamiento ortodóntico, o con evidente necesidad de este, individuos con restauraciones o prótesis fija que involucren caninos mandibulares, con enfermedad sistémica o local que afecte la estructura dental de caninos inferiores, así como aquellos estudiantes con ausencia de dichos dientes.

Luego de obtener el consentimiento informado de las unidades muestrales, se procedió a la toma de impresiones del maxilar inferior con alginato, para luego realizar el vaciado y obtener modelos de yeso piedra tipo IV. Una vez codificados tales modelos, se procedió a eliminar las piezas dentarias vecinas, con el objetivo de tener una mejor medición del ancho mesiodistal. Para la medición de los componentes del IMC se utilizó un vernier digital de 0-152,4 mm/0-6", de la marca Truper®, con una resolución de 0,01 mm. Los puntos más salientes de la cara mesial y distal de los caninos mandibulares se tomaron como referencia para el diámetro mesiodistal máximo, en tanto que para la distancia intercanina se consideró la punta de las cúspides de dichas piezas.

La fiabilidad del examinador se obtuvo al comparar, en 20 modelos de estudio, las mediciones del investigador con las realizadas por un perito experto. La prueba piloto obtuvo un valor CCI para medidas promedio de 0,912, lo que ratifica la confiabilidad del investigador para obtener los valores del IMC.

A efectos de determinar la eficacia se empleó el área bajo la curva (AUC), y se clasificó la exactitud de la prueba del siguiente modo: si el valor del área está comprendido entre 0,5 y 0,7, entonces la exactitud es baja; si está comprendido entre 0,7 y 0,9 la exactitud es regular-alta; y si es superior a 0,9, la exactitud de la prueba es alta. Para obtener el punto de corte discriminante de sexo en la población estudiada se utilizó el siguiente algoritmo:




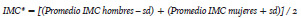




Para comprobar la distribución normal de las variables se empleó la prueba de Kolmogorov-Smirnov, mientras que para muestras no pareadas se utilizó la prueba de la T-Student con el fin de comparar los anchos mesiodistales, y para la distancia intercanina se consideró significativo un valor de p<0,05.

El valor diagnóstico del IMC se evaluó mediante la curva ROC *(receiver operating characteristic curve),* de modo que el canino que presentaba mayor área bajo la curva (AUC) discriminaba mejor entre hombres y mujeres. La curva ROC se calculó con el método no paramétrico de Hanley y Mcneil, que constituye un método estadístico para determinar la exactitud de test diagnósticos con escala continua, y que se utiliza con tres propósitos específicos: determinar el punto de corte de una escala continua en el que se alcanzan la sensibilidad y la especificidad más altas; evaluar su capacidad discriminativa del test diagnóstico, es decir, su capacidad de diferenciar sujetos sanos de sujetos enfermos, o, en nuestro caso particular, diferenciar hombres de mujeres; y comparar la capacidad discriminativa de dos o más test diagnósticos que expresan sus resultados como escalas continuas. El valor seleccionado como punto de corte fue aquel que tenía la mayor exactitud (mínimo número de resultados falsos negativos y falsos positivos).

## RESULTADOS

En el presente estudio se examinaron 168 estudiantes universitarios, de los cuales el 50 % pertenecieron al sexo masculino y el 50 % restante al sexo femenino. Luego de realizar los procedimientos clínicos descritos se obtuvieron los resultados que se presentan a continuación.

En la evaluación del grado de efectividad del IMC para la estimación del sexo, comparamos el IMC obtenido utilizando el canino izquierdo con el IMC obtenido utilizando el canino derecho. Se observa que el porcentaje de aciertos con el canino izquierdo fue de 25 % para el sexo masculino y 97,6 % para el sexo femenino; asimismo, el valor del área bajo la curva ROC mostró un resultado de 0,613, lo cual refleja un nivel de eficacia bajo para la toma de decisiones. Al utilizar el canino derecho, los resultados muestran que el porcentaje de aciertos para el sexo masculino fue de 28,6 % y para el sexo femenino de 98,8 %, mientras que el área bajo la curva ROC fue 0,637. El estadístico de Hanley y Mcneil establece que las diferencias de las áreas bajo la curva (eficacia para la estimación de sexo) no son estadísticamente significativas, con similares eficacias al utilizar el canino izquierdo y el derecho (p>0,05) (Tabla 1).

**Tabla 1:** Eficacia del IMC para determinación de sexo

H			Sexo IMC						
			Canino derecho			Canino izquierdo			P-Value
			MASC	FEM	AUC	MASC	FEM	AUC	
Sexo biológico	Masc Fem Total	N	21	63	0,613	24	60	0,637	0,6215
		%	25	75		28,61	71,40		
		N	2	82			83		
		%	2,40	97,60		1,20	98,80		
		N	23	145		25	143		
		%	13,70	86,30		14,90	85,10		

Prueba Hanley y McNei; p<0,05.

Luego de verificar la normalidad de los componentes del IMC (Kolmogorov-Smirnow; p>0,05), se procedió a comparar los anchos mesiodistales de caninos mandibulares homolaterales por sexo, y se pudo establecer que estos dientes funcionan como factores discriminantes de sexo. El promedio del ancho mesiodistal máximo en los caninos izquierdos fue de 7,18 mm en hombres y 6,81 mm en mujeres (p<0,05). Asimismo, al comparar los diámetros máximos de los caninos mandibulares derechos por sexo, se obtuvo que la media en los hombres fue de 7,21 mm y en las mujeres de 6,82 mm (p<0,05) (Tabla 2).

**Tabla 2:** Comparación del promedio del diámetro mesiodistal del canino mandibular izquierdo y derecho, según el sexo

Pieza dentaria	Sexo biológico	N	Promedio	Sd	*P-Value*
Canino izquierdo	Masculino	84	7,183	0, 413	9,76e-10
	Femenino		6,806	0,301	
Canino derecho	Masculino	84	7,210	0,419	2,47e-10
	Femenino		6,816	0,286	

Prueba T-Student independientes; p<0,05.

Al comparar los diámetros mesiodistales de los caninos mandibulares por lateralidad, los resultados muestran que las medidas de los promedios de los caninos derechos e izquierdos son similares en hombres y mujeres (p>0,05). El promedio del ancho mesiodistal del canino en el sexo masculino fue de 7,18 mm el izquierdo y 7,20 mm el derecho. De la misma manera, los caninos femeninos presentaron un promedio de 6,81 mm y 6,82 mm para los lados izquierdo y derecho, respectivamente (p>0,05) (Tabla 3).

**Tabla 3:** Comparación del promedio del diámetro mesiodistal de los caninos mandibulares por lateralidad

Diámetro mesiodistal	Lateralidad	N	Promedio	Sd	*P-Value*
Canino masculino	Izquierdo	84	7,183	0,413	0,684
	Derecho		7,210	0,419	
Canino femenino	Izquierdo	84	6,806	0,301	0,815
	Derecho		6,816	0,286	

Prueba T-Student independientes; p<0,05.

Los resultados de la comparación de la distancia intercanina en hombres y mujeres muestran que esta magnitud sirve como factor discriminante de sexo, puesto que los hombres obtienen en promedio 27,10 mm entre las cúspides de los caninos mandibulares, mientras que las mujeres alcanzan el valor de 26,84 mm. Las diferencias son estadísticamente significativas (p<0,05) (Tabla 4).

**Tabla 4:** Comparación de los promedios de las distancias intercaninas mandibulares, según el sexo

Sexo biológico	N	Promedio	SD	*p-value*
Masculino	84	27,101	0,743	0,027
Femenino		26,841	0,763	

Prueba T-Student independientes; p<0,05.

Al utilizar el algoritmo para punto de corte propuesto por Rao *et al.*^10^, que utiliza los promedios y las desviaciones estándar del IMC, se determinó el punto de corte tanto para el canino izquierdo (0,258) como para el derecho (0,259), y el nuevo punto de corte general obtenido con el promedio de ambos (0,258) (Tabla 5). Finalmente, se determinó la efectividad del nuevo IMC.

**Tabla 5:** Establecimiento de un nuevo punto de corte, discriminante de sexo, para la población de estudio

	Sexo biológico	Media	N	Desviación estándar	Punto de corte	Nuevo valor del IMC
IMC derecho	Masculino	0,26599	84	0,012809	0,2588365	0,258588.
	Femenino	0,25395	84	0,010542		
	Total	0,25997	168	0,013161		
IMC izquierdo	Masculino	0,26486	84	0,012276	0,2583395	
	Femenino	0,25369	84	0,010405		
	Total	0,25927	168	0,012652		

Prueba Hanley y McNei; p<0,05.

Los resultados muestran que la capacidad del nuevo IMC con el canino izquierdo de detectar a los hombres como hombres es del 73 %, y de identificar mujeres del 75 %>; el AUC es de 0,738, es decir, que establece la efectividad del nuevo punto de corte para el canino izquierdo como regular-alto. Los valores encontrados para el canino derecho fueron de 70,2 % de acierto para la detección de hombres y de 69 % para la identificación de mujeres; el valor AUC es de 0,7, lo cual lo clasifica como regular-alto; estadísticamente, la efectividad de ambos caninos es similar. Por último, el valor final de efectividad del punto de corte (0,258) es del 71,7 %. (p<0,05) (Tabla 6).

**Tabla 6:** Eficacia del IMC con el nuevo punto de corte para determinación de sexo

			Sexo IMC						
			Canino derecho			Canino izquierdo			P-Value
			MASC	FEM	AUC	MASC	FEM	AUC	
Sexo biológico	Masc	N	61	23	0,738	59	25	0,696	0,717
		%	73	27		70,2	29,8		
	Fem	N	21	63		26	58		
		%	25	75		31	69		
		N	82	86		85	168		
	Total	%	48,8	51,2		50,6	49,4		

Prueba Hanley y McNei; p<0,05.

## DISCUSIÓN

La identificación en cadáveres es una tarea complicada cuando solamente existen fragmentos óseos. Si bien es cierto que existen pruebas fehacientes como el ADN, estas a veces no están disponibles, por falta de equipamiento, accesibilidad o el factor costo, que en el Perú es un limitante importante. En estas circunstancias, los métodos de identificación odontoestomatológicos constituyen una alternativa importante para este fin 17.

Entre los dientes permanentes, los caninos son los que presentan mayor información con respecto a dimorfismo sexual; los caninos mandibulares permanentes se consideran dientes clave para fines de identificación, puesto que están menos expuestos a enfermedad periodontal, abrasión o carga oclusal pesada; por lo tanto, son de los últimos en ser extraídos con respecto a la edad. Además, pueden estar fácilmente disponibles, ya que el maxilar inferior es el hueso más fuerte del cuerpo humano y persiste en un estado bien conservado por más tiempo que cualquier otro hueso 11.

Uno de los principales factores reconstructivos de identificación es el sexo; asimismo, constituye la base para otros factores como la edad o la talla. En el presente estudio se pudo determinar la eficacia del IMC propuesto por Rao *et al.*^10^ para la estimación del sexo en una población del norte del Perú; igualmente, se encontró un nuevo punto de corte aplicable a nuestra población, con el que se aumenta la eficacia del IMC en aproximadamente un 10 %, puesto que pasamos del 62,2 % de eficacia al 71,7 %. El estudio fue elaborado luego de considerarse que hay diferencias en las características odontométricas en poblaciones específicas, incluso dentro de la misma población; en consecuencia, es necesario determinar valores específicos regionales para que sea posible la identificación con base en mediciones dentales 9.

El nuevo IMC encontrado en nuestra población fue de 0,258; es notorio que la mayoría de los estudios posteriores a Rao *et al.*^10^ encuentran puntos de corte inferiores al propuesto inicialmente. Singh *et al.*^14^ e Iqbal *et al.*^13^ coincidieron en que el nuevo punto de corte para sus poblaciones sería de 0,248, con el que registraron efectividades de 85,5 % y 77 %, respectivamente, las cuales son de similar efectividad que la alcanzada por Rao *et al.*^10^. De igual manera, Ankit *et al.*^11^ y Gandhi *et al.*^12^ estimaron los nuevos puntos de corte para sus poblaciones, de 0,254 el primero y 0,247 el segundo, y alcanzaron las correspondientes efectividades de 78,8 % y 79,03 %. Como concluyen Garn *et al.*^9^ en su estudio, los grupos étnicos presentan diferentes dimorfismos sexuales. En este contexto, cabe recordar que nuestro país posee un mestizaje bastante marcado, que incluye población autóctona con influencia de conquistadores europeos, migraciones asiáticas y esclavos africanos 18; por ello, no encontramos razas puras. Dada la variedad racial, es complicado, como sí se logra en los estudios anteriores, encontrar un punto de corte que englobe a un mayor número de personas; por consiguiente, se cree que el punto de corte discriminante hallado para la población de estudio (0,258) tiene una efectividad menor a los puntos de corte encontrados en otras poblaciones (71,7 %).

Cuando utilizamos el IMC de 0,274, propuesto inicial-mente por Rao *et al.*^10^, la eficacia de este método en nuestra población fue de 62,15 %, con una sobrepoblación de falsos positivos de mujeres, en desmedro de la población masculina. Esta frecuencia es similar al 63,8 % encontrado por Azevedo *et al.* y se encuentra muy por debajo del 73 % de Rajarathnam *et al.*^3^ y del 79,7 % de Ramniwas *et al.*^6^. Todos estos autores, en mayor o menor grado, manifiestan estar de acuerdo con que el IMC es una herramienta importante en la toma de decisiones con respecto al sexo.

Existen dudas por parte de los forenses con relación a qué canino tomar para la realización del IMC. El presente estudio encontró una ligera ventaja al utilizar el canino mandibular izquierdo (73,8 % de efectividad vs. 71,7 % del canino derecho). Sin embargo, al aplicar la prueba estadística estas diferencias son despreciables (p > 0,05); por ende, cualquiera de los caninos es válido para el IMC. Diferentes autores comparten similares resultados 3,6,19.

Por otro lado, se estimó la capacidad discriminante del sexo de los componentes del IMC, es decir, el ancho mesiodistal y la distancia intercanina. Existen claras diferencias (p < 0,05) entre los promedios de los diámetros mesiodistales de caninos mandibulares de hombres y de mujeres, lo que constituye, de esta manera, un potente predictor de sexo. Incluso autores como Azevedo *et al.*^2^ sostienen que los caninos son mejores discriminantes del sexo que el propio IMC. Rajarathnam *et al.*^3^, Kumawat *et al.*^6^ y Rani 19 corroboran las diferencias estadísticas y la potencia discriminante de sexo del canino mandibular.

El dimorfismo sexual puede atribuirse a muchos factores, que incluyen los de índole ambiental y los hábitos alimenticios. La teoría más aceptada es la de Eimerl y DeVore, quienes manifiestan que la apariencia de agresividad que proporcionan los caninos es una herencia genética de los primates de quienes evolucionamos. Estos dientes funcionaban como elemento disuasorio frente a los rivales y también como arma de ataque y defensa, principalmente en los machos, y por tal motivo constituían piezas de vital importancia para la supervivencia y la competencia por la reproducción 20. Las magnitudes halladas en el presente estudio corroboran el hecho biológico conocido que atribuye las diferencias en el grosor de esmalte a un período más largo de amelogénesis en el hombre. El dimorfismo sexual está controlado genéticamente. El cromosoma "Y", que determina el grosor de la dentina, interviene más en el tamaño de los dientes en comparación con el cromosoma "X", que tiene una mayor influencia en el grosor del esmalte 12,21.

En el presente estudio, la distancia intercanina también demostró constituir una magnitud discriminante de sexo. La diferencia de los promedios de las distancias intercaninas fue de 0,26 mm, que pese a ser un valor absoluto mínimo, es suficiente a nivel estadístico (p<0,05) para establecer diferencias.

No existen diferencias del IMC en cuanto a la lateralidad del canino escogido. Al comparar los anchos mesiodistales de los caninos mandibulares, las diferencias son despreciables, por lo cual, en el presente estudio se puede concluir que la medición de un lado puede ser representativa cuando no se pueda obtener la medición del otro lado. Por el contrario, Ghandi *et al.*^12^ manifiestan que el canino mandibular izquierdo presenta un mayor dimorfismo que el derecho. Cabe precisar que el algoritmo utilizado para dimorfismo dental incluye la fracción del ancho mesiodistal en hombres sobre la misma magnitud en mujeres; a este valor se le resta la unidad y se multiplica por 100. Los valores encontrados por Ghandi *et al.*^12^ fueron de 6,85 para el derecho y 7,62 para el izquierdo; nuestro estudio revela que los valores de dimorfismo son de 5,59 y de 5,80, respectivamente, para canino derecho e izquierdo. Si bien es cierto que el valor aritmético es mayor en el canino derecho, la diferencia no es significativa a nivel estadístico (p>0,05). Estas diferencias son una prueba clara del hecho de que la magnitud del dimorfismo sexual de los dientes caninos varía entre los diferentes grupos étnicos.

Con respecto al método, las mediciones con vernier digital mostraron una alta precisión y reproducibilidad, las cuales lo confirman como un instrumento adecuado para este tipo de investigaciones. La utilización de modelos de yeso, en lugar de la odontometría clínica, facilita la medición de los dientes y disminuye el riesgo de infección cruzada. Además, permite hacer múltiples mediciones a efectos de la calibración o la repetición de procedimiento, en casos de dudas o errores de medición causados por la fatiga del investigador.

El IMC es un método fácil, práctico y de utilidad para muestras numerosas y cuando otros medios no se encuentran disponibles, puesto que no requiere materiales de gran costo o aparatos sofisticados. La efectividad del IMC para la estimación de sexo, con el punto de corte estimado para la población del norte del Perú de 0. 258, es del 71,7 %. Este porcentaje sirve para la toma de decisiones, sin embargo, se recomienda complementar con otros medios odontológicos basados en odontometría y osteometría, puesto que la certeza no es absoluta ♠
